# Transgene-free genome editing and RNAi ectopic application in fruit trees: Potential and limitations

**DOI:** 10.3389/fpls.2022.979742

**Published:** 2022-10-17

**Authors:** Satyanarayana Gouthu, Christian Mandelli, Britt A. Eubanks, Laurent G. Deluc

**Affiliations:** ^1^ Department of Horticulture, Oregon State University, Corvallis, OR, United States; ^2^ Oregon Wine Research Institute, Oregon State University, Corvallis, OR, United States

**Keywords:** gene silencing and editing, RNP delivery, RNA-based ectopic application, fruit trees, genetic tools

## Abstract

For the past fifteen years, significant research advances in sequencing technology have led to a substantial increase in fruit tree genomic resources and databases with a massive number of OMICS datasets (transcriptomic, proteomics, metabolomics), helping to find associations between gene(s) and performance traits. Meanwhile, new technology tools have emerged for gain- and loss-of-function studies, specifically in gene silencing and developing tractable plant models for genetic transformation. Additionally, innovative and adapted transformation protocols have optimized genetic engineering in most fruit trees. The recent explosion of new gene-editing tools allows for broadening opportunities for functional studies in fruit trees. Yet, the fruit tree research community has not fully embraced these new technologies to provide large-scale genome characterizations as in cereals and other staple food crops. Instead, recent research efforts in the fruit trees appear to focus on two primary translational tools: transgene-free gene editing *via* Ribonucleoprotein (RNP) delivery and the ectopic application of RNA-based products in the field for crop protection. The inherent nature of the propagation system and the long juvenile phase of most fruit trees are significant justifications for the first technology. The second approach might have the public favor regarding sustainability and an eco-friendlier environment for a crop production system that could potentially replace the use of chemicals. Regardless of their potential, both technologies still depend on the foundational knowledge of gene-to-trait relationships generated from basic genetic studies. Therefore, we will discuss the status of gene silencing and DNA-based gene editing techniques for functional studies in fruit trees followed by the potential and limitations of their translational tools (RNP delivery and RNA-based products) in the context of crop production.

## Introduction

Fruit trees are an essential specialty crop, part of the worldwide food production and economic system, representing at least ~750 M metric tons produced in 2020 (www.fao.org). Conventional breeding has ensured for decades the improvement of consumer-driven traits, including yield, size, nutritional properties, aroma, taste, and the introduction of agronomic characteristics, like tolerance to abiotic and biotic stress. Even with modern molecular approaches, breeding is slow due to the long juvenile phase of most fruit tree species, and the heterozygous nature of the varieties prevents them from maintaining the integrity of their original genetic makeup without several cycles of crosses. Therefore, conventional breeding may not be the most efficient approach to rapidly developing new varieties to meet the challenges of evolving climate, “volatile” consumer preferences, and other changing socio-economic factors such as decreasing labor force and energy costs.

While the recent increase in fruit tree crop genomic resources and database availability is regarded as a significant trigger to improving the understanding of gene function, recent advances in advanced biotechnology tools like RNAi-based gene silencing and gene editing are of paramount importance to accelerating gene function studies beyond the gene-to-traits associations inferred from most “OMICS” technologies. For the past twenty years, significant signs of progress have been made in most fruit trees for reverse and forward genetics programs (Peña et al., 2004; Malnoy et al., 2009; Chaïb et al., 2010; Petri et al., 2018; Savadi et al., 2021). Thanks to more precise and advanced genetic systems, the functional characterization of key genes to essential performance traits in fruit trees is rapidly increasing. Yet, there is still a significant gap in the amount of scientific information generated from fruit trees compared to other major crops that will incite the development of more translational and sustainable technology to respond to immediate needs.

In the first section of this review, we will summarize the most recently advanced tools, RNAi-based gene silencing and gene editing *via* DNA-targeting Cas effectors that could be exploited to advance fundamental knowledge on the gene(s) to trait associations for primary fruit and vine trees (apple, grape, pear, citrus, kiwifruit, and prunus). A few examples from recent literature will showcase the current knowledge of fruit trees. In the following two sections, we will discuss the emerging development of transformative tools that are gaining public and scientific traction: Ribonucleoprotein delivery and ectopic application of RNA in plants. We will cover the recent advances in both technologies, their potential, their limitations, and the major scientific priorities that need to be addressed for these tools to become efficient and transformative in fruit trees for improved crop production.

## Current status of gene silencing and DNA-based gene editing tools for fruit trees

**Figure 1:**
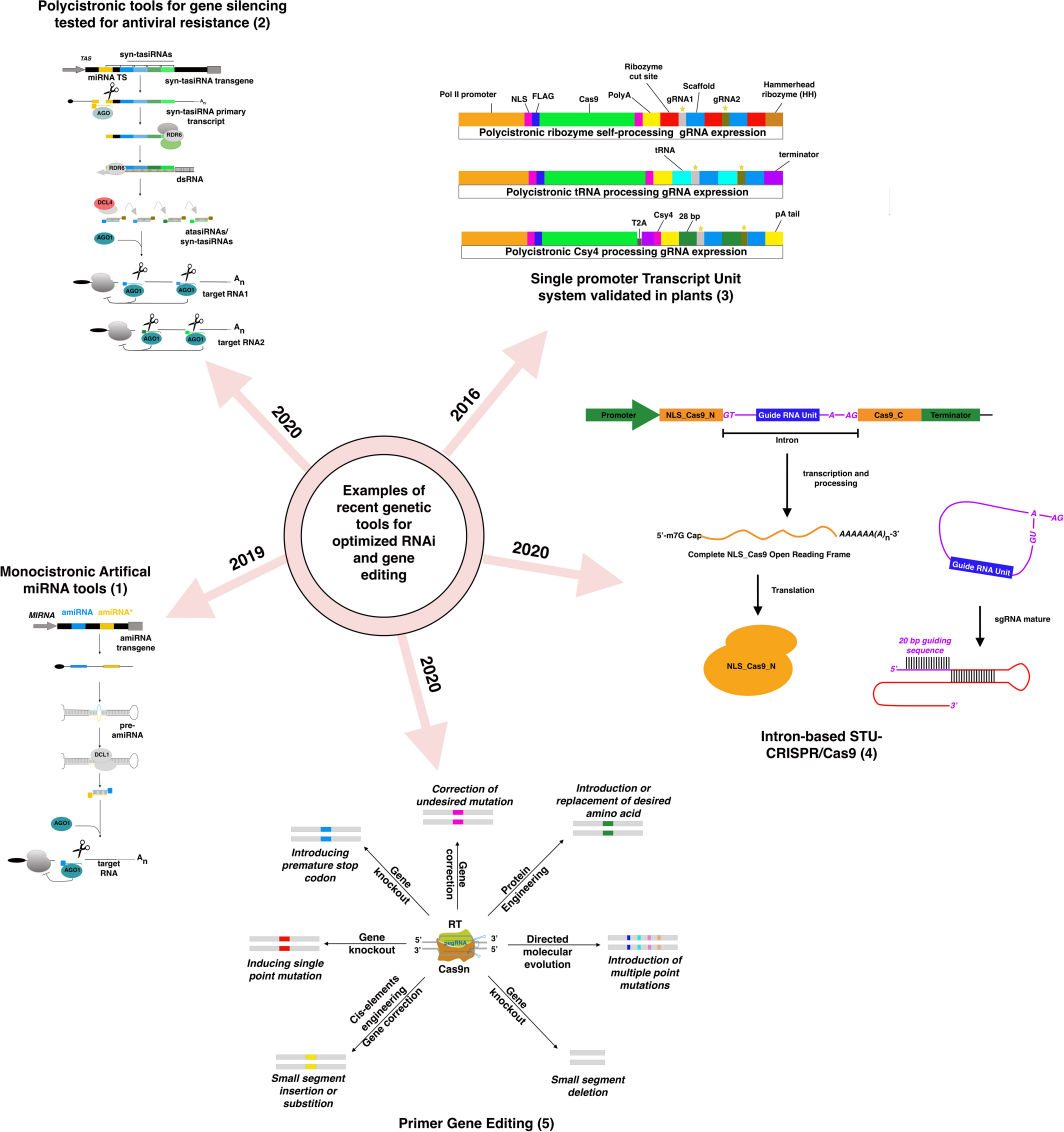
Examples of recently developed and validated genetic tools in plants for RNAi-silencing and DNA-based gene editing based on figures from previously published figures: (1) (Carbonell, 2019), (2) (Cisneros and Carbonell, 2020), (3) (Lowder et al., 2016), (4) (Zhong et al., 2020), (5) (Hassan et al., 2020).

Gene silencing involves suppressing gene expression by either repressing its transcription (Transcriptional Gene Silencing or TGS) or influencing the mRNA expression or the protein level, known as Post-Transcriptional Gene silencing (PTGS). Several tools for PTGS and TGS based on hairpin RNAs (hpRNA), trans-acting small Interfering RNAs (tasiRNA), and microRNAs have been developed over the past ten years. Gene silencing based on hairpin remains the most popular, with improved versions like introducing an intron between the RNA arms to enhance the stability of the hpRNA (Wulfert and Krueger, 2018). Viral-Induced Gene Silencing (VIGS), based on a modified virus containing a fragment of a gene to be silenced, leads to the production of double-stranded RNAs (dsRNA) complementary to the target gene. MicroRNA-induced gene silencing (MIGS) introduced the multiplexing approach to target multiple related or unrelated genes (Han et al., 2015). The development of artificial microRNAs (amiRNA)-based gene silencing, based on the expression of custom primary microRNAs, opened new opportunities for a broader targeting (Carbonell et al., 2014). Like MIGS, artificial or synthetic tasiRNAs (atasiRNAs and syn-tasiRNAs) operate through the action of secondary siRNAs that induce selective gene silencing (**Figure 1**) (Cisneros and Carbonell, 2020). The syn-tasiRNAs construct expressing different syn-tasiRNAs from a single precursor is a potent tool to target multiple viral RNAs. The simultaneous expression of several syn-tasiRNAs against Tomato spotted wilt virus (TSWV), an economically harmful pathogen in tomato crops worldwide, resulted in strong resistance against the virus in all generated transgenic lines (Carbonell, 2019). These last systems were proven effective in monocots and major fruit crops like tomato, but few studies expanded their use to fruit tree (Charrier et al., 2019b). These performant genetic tools, in conjunction with the rapidly increased implementation of machine learning tools, could exponentially increase the identification of efficient siRNA species with greater on-target efficacy and fewer off-target risks in fruit trees models (Wang et al., 2010; Fahlgren et al., 2016; Ahmed et al., 2020). Yet, in recent years, few of these tools have been applied to fruit trees studies, except VIGS, sense-gene-induced post-transcriptional gene silencing (S-PTGS) approaches (Liu et al., 2018; Qi et al., 2019; Singh et al., 2019; Werner Ribeiro et al., 2020) and RNAi-based vector systems that generated a hairpin structure (Pessina et al., 2016; Li et al., 2020; Huang et al., 2021; Wu et al., 2021). To the best of our knowledge, no functional gene studies have explored the advantages of amiRNAs and syn-tasiRNAs studies in fruit trees with the exception of Charrier’s study (Charrier et al., 2019b), while polycistronic amiRNA and syn-tasiRNAs tools have demonstrated their efficiency in creating antiviral resistance (Carbonell et al., 2019; Miao et al., 2021). The lack of robust and tractable genetic systems in fruit trees, in conjunction with the explosion of the gene-editing multifaceted technology, could potentially explain this lack of willingness to adopt more performing and higher throughput RNAi-based technologies for knock-down generation.

The gene-editing technology, as it is, already offers more versatile tools than RNAi-based gene silencing for multiplexed targeting. Implementing sgRNA arrays within the same construct simultaneously targeting up to 12 genes offers more significant opportunities for high throughput screening of mutants (Tang et al., 2016; Stuttmann et al., 2021). If the uncoordinated expression of the two components of the editing system (Cas protein and the guide RNAs) had been a significant drawback at the beginning of the editing era, the progressive adoption of Single Transcript Unit systems has radically improved the editing efficiency and the versatility of the tools regardless the targeted crops (Tang et al., 2016; Tang et al., 2019; Zhong et al., 2020). Identifying new Cas9 and Cas12a with different PAM requirements has expanded the range of DNA recognition for the broader-targeted gene editing (Tang et al., 2017; Wang et al., 2017a; Zhong et al., 2018). Precise genome editing techniques have suffered from conceptual pitfalls for years. Recent technology advances have attempted to address major bottlenecks. The engineering of the genetic cassette enabling for local presence of the donor template near the cut site was found to improve the editing efficiency rate (Ali et al., 2020). Using CRISPR base editors (a modified Cas9 with a cytosine or adenine deaminase domain) is regarded as a promising and exciting alternative to avoid donor templates. Still, the catalog is currently limited to C-to-T and A-to-G base conversion (Komor et al., 2016). To overcome this limitation, a new type of primer Editors was developed, and based on chimeric nCas9 protein fused to an M-MLV reverse transcriptase, a primer-editing guide RNA (pegRNA) designed to mediate site-specific nicking then serves as a template for RT (Anzalone et al., 2019). This system was successfully adopted for monocots and is likely to work for dicots (Butt et al., 2020; Lin et al., 2020; Xu et al., 2020).

To the best of our knowledge, most gene-editing studies were designed to create stable knockout *via Agrobacterium*-mediated transformation to infer association to major agronomic traits like flower and fruit architecture/composition, disease resistance, and for improving breeding purposes like in kiwi fruits (Varkonyi‐Gasic et al., 2019; Charrier et al., 2019a; Pompili et al., 2020; Wan et al., 2020; Iocco-Corena et al., 2021). All of them used the popular CRISPR-Cas9 system to achieve editing. A special note should be made on gene-editing technology to combat Citrus canker caused by the *Xanthomonas citri* subspecies a significant disease for *citrus* production worldwide (Vojnov et al., 2010). Through several studies (Hu et al., 2014; Jia et al., 2016; Jia et al., 2017), one biallelic mutant of the CsLOB1 promoter region involved in the interaction with the bacterial TALE (transcription activator-like effector) was found to confer to ‘Duncan’ grapefruits complete immunity to *Xanthomonas* (Jia et al., 2022). In other fruit crops beyond fruit trees, gene editing including in strawberry (Zhou et al., 2018), banana (Kaur et al., 2018), and watermelon (Tian et al., 2018), were all through stable transgenic transformations.

The recent gene-edited crops approved by USDA also used stable expression through *Agrobacterium*-mediated transformation or particle bombardment but were followed by the segregation of transgenes through selfing and crossing (Lacroix and Citovsky, 2020; Gao, 2021) to generate transgene-free plants, which is not possible in clonal fruit crops. Direct modification of crop genomes to introduce economically essential traits without GMO labels has strengthened plant breeding efforts. USDA views the crop varieties developed through genome editing technology as the products of plant breeding as long as no foreign DNA is inserted into the genome (“USDA APHIS | Regulated Article Letters of Inquiry”). This encouraged the development of transgene-free approaches to introduce agronomic traits, and the number of gene-edited crops approved by the USDA jumped from 7 to 70 from 2019 to 2020 (Bomgardner, 2020). Country-wide status of the regulatory, and legislative status towards gene-edited crops has been well discussed in the review (Turnbull et al., 2021). Yet many clonally propagated fruit crops did not benefit from this technology mostly because of the recalcitrance of these crops to have gene-editing reagents delivered into regenerable plant materials without the need for further crossings.

Similarly, technologies based on ectopic RNA application to induce RNAi have also gained significant traction due to the non-GMO nature of the technology (Taning et al., 2021). Currently used for plant protection against fungal pathogens and pests, the technology is an alternative to conventional pesticides for more sustained production systems. RNAi-based products offer multiple advantages compared to their chemical counterparts (Taning et al., 2020). First, the dsRNA active molecules can be designed to target the expression of different genes without changing the sequence-dependent mode of action. Secondly, the availability of increasingly robust *in silico* tools for dsRNA design, in conjunction with the growing access to genomic resources, makes it possible to design species-specific molecules with negligible off-target effects compared with current broad-spectrum pesticides with undesirable side-effects. dsRNA molecules can be rapidly degraded, limiting their long-term environmental persistence. RNAi-based biocontrol delivered by exogenous application of formulated RNA molecules might have the public favor because plants treated with exogenous dsRNA are not considered genetically modified organisms (Shew et al., 2017). Finally, when one compares transgenic approaches, the fast and temporary use of this technology during specific times of the growing season may also offer more leverage, versatility, and reactiveness in the number of applications and the nature of applied materials. There is an increasing number of studies reporting RNA-based product applications in many crops, including fruit trees, but all were performed in a laboratory setting. The recent study conducted by Wise et al., 2022 (see below) is encouraging, but RNAi technology’s applicability to field conditions may vary from a given crop production system to another. Multiple limiting factors exist in a field setting, including overcoming the plant’s physical barriers for uptake, the effects of environmental factors on the extent of RNA silencing, and achieving systemic silencing to the whole plant. Altogether, this will need to be addressed to maximize the scalability and processibility of the technology in a crop production system.

## RNP delivery: Applications and limitations

Besides eliminating transgenes after generating transgenic gene-edited plants through conventional stable transformation methods, direct transgene-free editing can be performed through either transient expression of plasmids or directly using CRISPR elements as ribonucleoproteins. Transient expression of CRISPR elements without integration into the plant genome has been reported in the grapevine using a Geminiviral replicon system (Olivares et al., 2021). But direct gene editing using CRISPR elements as ribonucleoproteins (RNP), without the use of DNA, first demonstrated by (Woo et al., 2015), is the most promising and desirable option to generate transgene-free plants because it avoids DNA insertions and accomplishes gene editing in one generation without unwanted crossings in most clonal crops. However, the success of this technique in fruit crops depends on two main factors: cellular delivery of RNPs and identification of edited material in the absence of selection markers, which are discussed below.

Delivery of CRISPR RNPs is a significant challenge in plants because standard transfection techniques used in animals are typically ineffective in intact plant cells. The genome editing reagents can be delivered into protoplasts without cell walls *via* polyethylene glycol (PEG)-mediated transfection. Therefore, protoplast transfection is commonly used in model organisms and many crops to demonstrate the efficiency of RNP-mediated gene editing. PEG-mediated RNP delivery into protoplasts has been performed in many plant species such as Arabidopsis, rice, lettuce, tobacco (Woo et al., 2015), petunia (Yu et al., 2021), maize (Sant’Ana et al., 2020), wheat (Liang et al., 2017), soybean (Kim et al., 2017), potato (Andersson et al., 2018), cabbage (Murovec et al., 2018), including fruit crops of grapevine, apple (Malnoy et al., 2016), and banana (Wu et al., 2020). These studies reported editing efficiencies in regenerated microcalli, shoots, or plants in the low 11% in petunia to 25% in the potato model. Besides cellular internalization of RNPs and successful editing, the editing efficiency estimates largely depend on protoplast regeneration efficiency, which remains very low in some species or impossible in many fruit crops. Following PEG-mediated protoplast transfection, gene-edited plants were regenerated in some plant models, such as *N. benthamiana* and *B. oleracea* (Woo et al., 2015; Lin et al., 2018; Hsu et al., 2021). In fruit crops, including grapevine, apple, and citrus, these studies were limited to demonstrating RNP-mediated transgene-free gene-editing technique (Malnoy et al., 2016; Zhang et al., 2022b) mainly due to the challenges in regenerating plants from protoplasts. In this regard, the recent establishment of protoplast regeneration protocols in banana, grapevine, guava, and oil palm holds a lot of promise (Reed and Bargmann, 2021). Still, an ideal delivery method for fruit crops would be able to carry CRISPR RNPs and penetrate the cell wall and cell membrane into intact regenerable cells. To overcome the protoplast regeneration, CRISPR RNPs can be transfected *via* PEG transfection into zygotic cells of rice, taking advantage of the immature cell wall during the early zygotic period. The researchers achieved targeted mutations in 14-64% of plants (Toda et al., 2019). However*, in vitro* electro-fusion of isolated gametes is technically challenging for broader application, especially in clonally propagated crop species. On the other hand, transgene-free gene editing has been attempted through biolistic delivery of RNPs in immature wheat embryos and intact tobacco BY2 cells with 3-5% mutagenesis frequencies (Liang et al., 2017; Liu et al., 2020a). Other potential technologies being explored for RNP delivery include nanoparticles and the cell-penetrating peptides (Bilichak et al., 2020).

The cell wall, which makes RNP delivery challenging with current techniques, comprises a complex network of carbohydrates with a negative charge and allows only small molecules through. Studies that estimated the pore size of cell walls found that the cell wall size exclusion limit (SEL) was generally within the 5-20 nm range (Etxeberria et al., 2016; Cunningham et al., 2018). Once in the apoplastic space across the cell wall, the cell membrane has a much larger exclusion limit of 300-500 nm (Wang et al., 2019). So, to pass these two barriers, the RNPs and the carrier should be smaller than the cell wall SEL, and the carrier must have a motif enabling the plasma membrane passage likely *via* endocytosis. While the size of the Cas9 RNP is expected to be 7 to 9 nm, the size of the RNPs with carrier complexes might reach from 25 nm in the case of individually nanocapsuled Cas9 RNPs to 500 nm in case of aggregated nano assemblies that are much larger than the cell wall SEL (Mout et al., 2017a; Mout et al., 2017b; Chen et al., 2019). Biolistic delivery circumvents the cell wall SEL and cell membrane permeability issues through mechanical force. Most plant tissues are amenable to biolistic gene transfer but problems with strong cuticles, lignified cell walls, or hairy surfaces that resist particle penetration can occur. RNP-mediated genome editing using biolistic methods in intact tissues has been demonstrated in rice (Banakar et al., 2020), maize (Svitashev et al., 2016), and wheat (Liang et al., 2017; Liang et al., 2019; Liu et al., 2020b) with editing efficiency usually less than 10%. It was shown that the protein delivery of the Cas9/gRNA RNPs into plant cells had lower off-target cleavage rates when compared with the DNA-based delivery of the Cas9/gRNA complex (Svitashev et al., 2016; Zhang et al., 2016; Liang et al., 2017). The limitation of the biolistic approach to delivering CRISPR RNPs is the need to adapt particle bombardment protocols for each type of target tissue, which necessitates the adjustment of several critical variables such as particle diameter and distance from the target material (Lacroix and Citovsky, 2020) and low transformation frequency due to a small number of cells receiving microprojectiles (Banakar et al., 2019). Despite its low rate of delivery and possible integration of DNA fragments into the genome and genome-scale sequence disruptions (Zhang et al., 2016; Banakar et al., 2019; Liu et al., 2019), CRISPR RNPs delivery through biolistics is still a practical method in fruit crops. Biolistic RNP delivery into intact (Awasthi et al., 2022).

There are several reports of large biomolecules measuring >200 nm delivered across the cell wall in calli and intact plant tissues with the help of nanoparticle carriers and cell-penetrating peptides (CPPs) without forced biolistics (Ng et al., 2016; Guo et al., 2019), which is difficult to explain. Specific nanoparticles interact with the cell wall changing the pore sizes and formation of new pores (Asli and Neumann, 2009; Ma et al., 2010; Palocci et al., 2017) and preincubation with certain pro-endocytotic peptide carriers causing cell wall modifications were also reported (Wang et al., 2021a). Size dynamics of cell wall pores can also vary depending on cell type, degree of development, and physiological stage of the cell. Permeability to nanoparticles might increase in newly synthesized cell walls of actively dividing cells and cultured cells where the wall texture is less dense and less structurally organized (Navarro et al., 2008; Palocci et al., 2017). Assuming interaction occurs between the carrier particles and the biopolymers of the cell wall, the internalization of proteins bigger than the exclusion limit is plausible. Various nanoparticle platforms, including lipid nanoparticles, polymer-based nanoparticles, DNA nanoclews, and gold-based nanoparticles, have successfully delivered CRSPR RNPs across the cell membrane into human cell lines for the genome editing (Duan et al., 2021). However, there is no literature on nanoparticle-mediated RNP delivery for genome editing in walled plant cells, which could be a massive advantage for fruit crops to avoid the need for crossing and protoplast regeneration. Other potential tools for protein delivery into intact plant cells and tissues are cell-penetrating peptides (Ng et al., 2016; Guo et al., 2019; Midorikawa et al., 2019). A recent study by Numata’s group screened 55 CPPs, without protein cargo to determine the optimal CPP characteristics for penetration into the intact plant tissues of different species (Numata et al., 2018). The optimal composition of CPPs for the highest penetrating efficiency and nuclear localization differ across the plant species. Still, in general, Lys-containing CPPs seem to be more efficient for plant delivery (Numata et al., 2018) compared to Arg-rich peptides such as Tat peptide favored in animal cells (Taylor and Zahid, 2020; Trofimenko et al., 2021), which could be due to the differences in significant lipid components of their cell membranes. Generally, CPPs, similar to nanocarriers, are believed to internalize by classical endocytic pathways (Xie et al., 2020; Francia et al., 2022), but detailed studies are needed to clarify the differences. In animal systems, simple co-incubation is enough to internalize the proteins delivered through nanocarriers or CPPs. Due to the presence of the cell wall, mild infiltration force such as vacuum or centrifugal force is applied to deliver protein cargoes in plants without loss of regeneration efficiency (Kimura et al., 2019; Watanabe et al., 2021). CPPs have been used in animal cells to deliver genome-editing elements as RNPs non-biolistically, and these short, positively charged peptides were shown to translocate across cell membranes with high delivery efficiencies (Liu et al., 2014; Ramakrishna et al., 2014; Alghuthaymi et al., 2021; Gustafsson et al., 2021). In plants, nanocarriers and CPPs were used to deliver large proteins such as alcohol dehydrogenase (150 KDa) across the cell wall into intact plant tissues (Ng et al., 2016; Wang et al., 2021a). How these proteins attached to CPPs and nanomaterials much larger than cell wall SEL can pass through remains unanswered. Regardless of the mechanism, the ability of CPPs to deliver large proteins across the cell wall highlights their potential for transgene-free genome editing in clonal fruit plants.

Transgene-free gene editing, either through transient expression of plasmid DNAs or RNP delivery, does not afford selection in contrast to stable transformation with selectable marker genes. The reported transgene-free mutation rate in tobacco and wheat through transient expression or RNP biolistic techniques is currently low at 2.5 to 9% using explants and 0.5% cells while using calli (Svitashev et al., 2016; Chen et al., 2018a; Zhang et al., 2019b). When no selection pressure is applied during callus and shoot regeneration following these techniques, most regenerated embryos/shoots should be non-mutant. It should be followed by high throughput screenings such as sequencing, high-resolution melting analysis, etc. Approaches like selectable co-editing followed by Zhang et al., 2019b that confers herbicide tolerance *via* co-editing of acetolactate synthase gene is appealing but may not be applicable for all the crop species.

## Ectopic application of siRNA: A real opportunity for lab-to-field transitions in fruit trees?

Several factors like penetration, stability and diffusion of RNA molecules in plants must be considered to evaluate the ectopic application of RNAs as a promising tool in the field, especially for tree crops, due to size and field-practice constraints. dsRNA molecules can be delivered through different methods, including foliar spray, recombinant microbes, nanoparticles, trunk injection, and root soaking, with variable outcomes that rely on the plant model itself (Koch et al., 2016; Wang and Jin, 2017; Liu et al., 2021; Pugsley et al., 2021). To the best of our knowledge, there is currently one study reporting on the systemic effect of trunk injection-mediated delivery of dsRNA in fruit tree crops under field conditions (Wise et al., 2022). The two year-study clearly showed a gradual decline but persistence of dsRNA molecules in the tree canopies over the growing season following the treatment in the spring. The potential of dsRNA delivery has been extensively adopted and reported in many instances with pests and pathogens as a successful method for plant protection. However, with few exceptions showing significant results (Wang et al., 2016) against major pathogens for crop production like *Botrytis cinerea*, most silencing studies through RNA application were validated using transgenic materials that were over-expressing the transgenes (Dalakouras et al., 2016; Schwartz et al., 2020; Hendrix et al., 2021). Foliar application is another promising avenue for tree crops but suffers the same issues as RNP delivery regarding physical barriers to cross. The lipophilic nature of the cuticle hampers the absorption of exogenous hydrophilic and polar molecules like nucleic acids (Schreiber, 2005) that can be penetrated *via* an abrasion, high-pressure spraying, and abaxial stomatal flooding but the applicability of such treatment in a field setting is not realistic (Dalakouras et al., 2016). Beyond the cuticle, the dsRNA molecules’ size, length, and shape are essential determining factors in crossing the next physical barrier, the cell wall. It remains a significant hurdle for delivering long RNA molecules and even for a siRNA molecule that does not exceed 26 nucleotide long in many instances. With an averaged pore size exclusion of 6 nm, the size of dsRNA cannot exceed, in theory, more than 16 nm to cross the cell wall (Bennett et al., 2020; Kurczyńska et al., 2021). A recent study in tobacco BY-2 cells suggests a pore SEL for 90 bp long nucleic acids corresponding to 31 nm (Bennett et al., 2020), which seems to suggest that crossing the cell wall is instead a flexible and dynamic process, which can be potentially manipulated to a certain extent and for which the use of nanocarriers may play a critical role in increasing the uptake (Schwartz et al., 2020). The plasma membrane appears to be less problematic to be crossed as several studies have reported the internalization of RNA molecules as long as 500 nm (Wang et al., 2014). Still, it may depend on the shape of the nucleic acids (Zhang et al., 2022a).

The use of nanocarriers to deliver drugs, proteins, and nucleic acids has been widely exploited in the medical sciences (Zhang et al., 2022a). Their use in plant sciences remains highly potential but anecdotal for pest and pathogen control. Nanocarriers serve two purposes for ectopic application of dsRNA: protection and improved uptake (Šečić and Kogel, 2021). Several classes of nanocarriers/nanoparticles (NPs) were shown to protect and extend the integrity of RNA molecules at the leaf surface and inside the cells (Mitter et al., 2017). Numerous carriers have been tested for delivery to various plant models (Wang et al., 2021b; Zhang et al., 2022a). The proper class of NPs used for a given crop primarily depends on the delivery method (spray techniques, root drenching, or trunk injection). Additionally, other criteria need to be specifically evaluated in field conditions because plants may respond differently to a given nanocarrier depending on the plant’s architecture and environmental conditions. Some recently tested carriers include but are not limited to carbon nanodots (Schwartz et al., 2020), carbon nanotubes (CN) (Kwak et al., 2019; Demirer et al., 2020), Layered Double Hydroxides of clay (LDH) (Mitter et al., 2017), iron oxide compounds (Cai et al., 2020), multifaceted histidine-based nanocarriers (He et al., 2020), and cationic polymers mimicking Arginine-rich cell-penetrating peptides (CPP) (Parsons et al., 2018). Studies comparing naked RNA versus complexed ones to NPs have ascertained an improved foliar uptake with NPs (Mitter et al., 2017; Schwartz et al., 2020; Delgado-Martín et al., 2022).

Whether this improved uptake is associated with better RNA protection, hence limiting their degradation, or the NPs’ physical and chemical properties influencing the RNA’s internalization *via* endocytosis mechanisms is debatable (Zhang et al., 2022a). Layered Double Hydroxide molecules were found to increase the uptake of dsRNA molecules by directly maintaining their integrity on leave surfaces (Mitter et al., 2017). Finally, non-engineered and non-metal particles like DNA nanostructures are also capable of delivering exogenous biomolecules like siRNA because of their inherent biocompatibility with plant structural components. The reduced risk of phytotoxicity and traceability compared with conventional NPs renders their use even more attractive for sustainability purposes (Zhang et al., 2019a). Another aspect of the NP’s choice is their size. Carbon Dots (CDs), besides the simplistic and advantageous scalability of their synthesis, have an average hydrodynamic diameter of 2.6 nm, which is below the average SEL of the cell wall, even when combined with siRNA (4.7 ± 0.8 nm) (Wang et al., 2014). Interestingly, complexes including dsRNA instead of siRNA showed hydrodynamic diameters in the 160 to 350 nm range with an efficient RNA uptake (Schwartz et al., 2020; Ng et al., 2021; Delgado-Martín et al., 2022). These results again challenge the issue of crossing the cell wall wherein the SEL can be somewhat overcome depending on the employed NP’s physical and chemical characteristics.

Most efficient silencing experiments were observed with the ectopic application of 21-22 nucleotide siRNA instead of longer dsRNA regardless of the silencing extent (local or systemic) (Bennett et al., 2020). Thus, the selection of an effective siRNA sequence remains an important prerequisite for potent applications of RNAi-based products in the field as the identification of siRNA with high efficacy *via* genetic engineering remains largely dependent on genetic studies with stable transformations (Mitter et al., 2017; Schwartz et al., 2020; Delgado-Martín et al., 2022) or experiments targeting GFP expression (Dalakouras et al., 2018; Schwartz et al., 2020). The role of 22 nucleotide siRNA species in triggering a systemic RNAi in the whole plant is well documented. It requires the action of the RDR6 polymerase that is likely responsible for the amplification, the transitivity, and the systemic spread of RNAi (McHale et al., 2013; Taochy et al., 2017; Chen et al., 2018b). However, the recruitment of RDR6 can occur with any-sized sRNA that contains an asymmetric bulge in its duplex structure, which leaves open the opportunity to implement a silencing with a siRNA species other than 22 nucleotide long (Manavella et al., 2012; Dalakouras et al., 2016; Dalakouras et al., 2018). Therefore, coupling the increasing genomic resources of fruit trees to developing robust *in silico* tools to predict different classes of siRNAs species with a systemic silencing potential is a significant priority. Algorithms like Support Vector Machines (SVM) can be trained and tested over a sequence dataset, which has already been experimentally validated. Once the best prediction parameters are set up, they can be used to predict siRNAs from long dsRNA sequences. Sequence composition features have historically represented most of the critical features used in the SVM pipeline as di- and tri-nucleotide counts, global and local GC content, duplex flexibility, and thermodynamics stability (Shabalina et al., 2006; Sciabola et al., 2021).

Additionally, target accessibility and the 5’ siRNA composition to load into AGO proteins are critical to the silencing characteristics of a siRNA sequence (Gago-Zachert et al., 2019). *In silico* tools that strategize a hybrid-SVM-based prediction approach based on efficacy scores from previously designed models (nonspecific and toxic siRNAs removal, intended versus unintended target transcripts, RISC loading efficacy of the siRNA, target site accessibility, highly specific siRNA) combined with training datasets will generate a list of siRNA to be tested with higher confidence (Ahmed et al., 2020). Through this approach, Ahmed et al. (2020) identified new siRNA candidates targeting highly expressed gene (GFP gene) and endogenous genes in tobacco, Arabidopsis, and periwinkle with relatively high confidence (correlation coefficient greater than 0.7 between the measured predicted efficacy) of the siRNA candidate. Overall, using siRNAs could represent the most efficient way to overcome the plant’s physical barriers. Still, it requires significant progress in developing robust predicting tools to identify effective siRNA.

**Figure 2:**
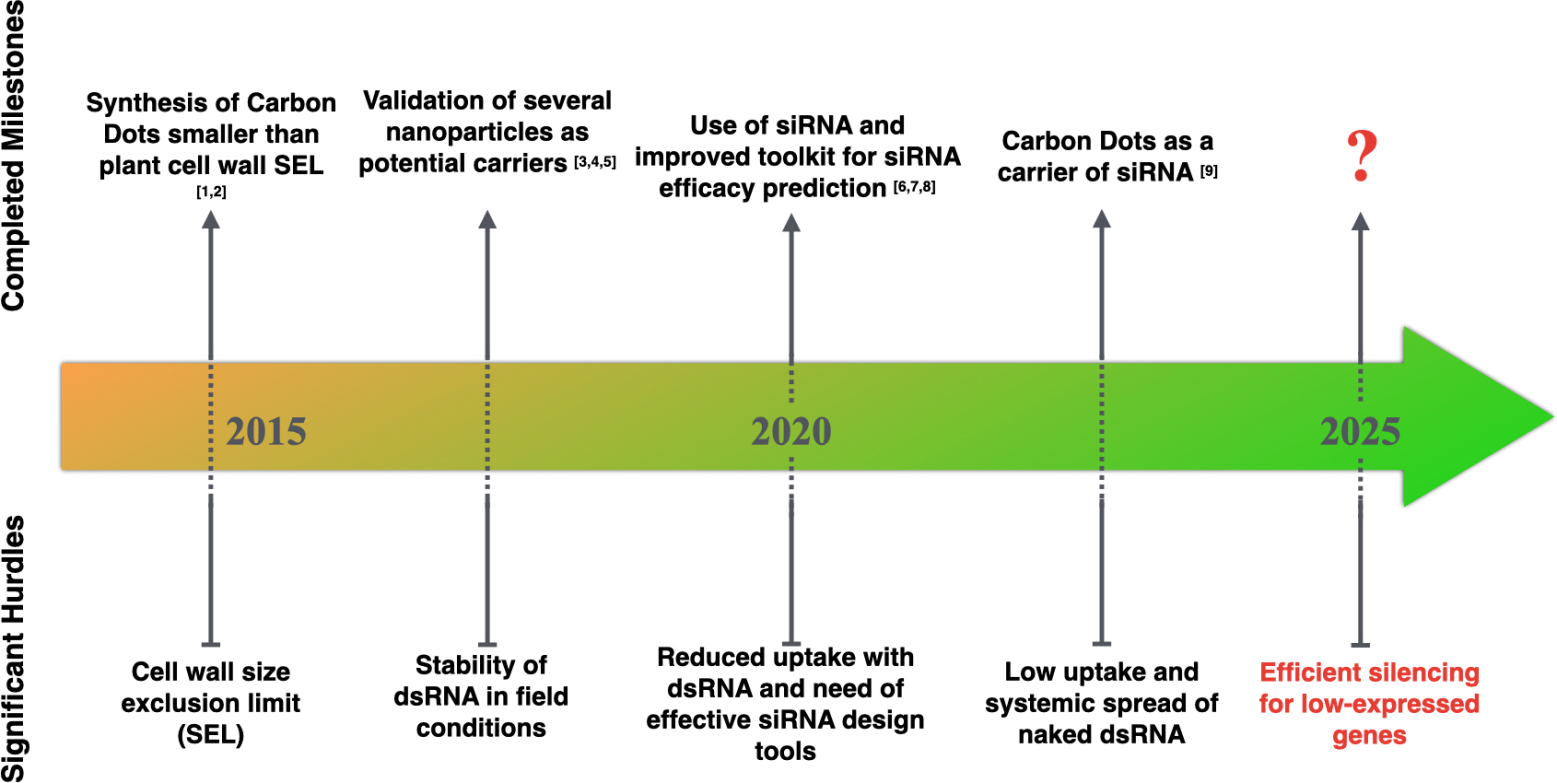
Significant milestones achieved for RNA-based ectopic application. [1, 2]: Wang et al., 2014; Schwartz et al., 2020, [3-5]: Mitter et al., 2017, Zhang et al., 2019a, Demirer et al., 2020, [6-8]: Ahmed et al., 2020, Bennett et al., 2020, Sciabola et al., 2021, [9]: Delgado-Martín et al., 2022.

Ultimately, the systemic component of silencing remains essential to assess the technology in a field setting. Optimizing systemic silencing within trees will reduce ectopic application costs to a minimum. The plant’s age could also be an important influencing factor. While young plants tend to be more susceptible to pathogens and pests due to less accumulation of waxy cuticles and trichomes, these unprotected portions may be more amenable to absorbing RNA-based products for better protection. When plant maturity is reached, both plant size and mature leaf structure become challenging for efficient absorption of sprayed RNAs, systemic spread, and the resulting silencing. Werner et al., 2020 found that the foliar application of small hairpin dsRNA effectively inhibited *Fusarium graminearum* infection in non-sprayed tissues of barley (*Hordeum vulgare*). By improving the RNA uptake, the NPs also are likely to impact the strength of secondary siRNA production and probably the extent of systemic spread of silencing. Schwartz et al., 2020 demonstrated that carbon nanodots bound to siRNA molecules silence target genes in both locally and newly emerging leaves. Mitter et al. (2017) found a more significant systemic movement of dsRNA-treated with LDH in non-treated parts of the cucumber and tobacco plants. Delgado-Martín et al. (2022) demonstrated greater efficiency of Carbon Dots to induce a systemic spread of dsRNA with even lesser dsRNA molecules applied, which could be advantageous in terms of synthesis cost. Though, very little data have demonstrated systemic silencing of endogenous genes, which brings uncertainty to the translation of the technology, especially to control low expressed endogenous genes (Marcianò et al., 2021; Nerva et al., 2022). There is still a substantial work to determine whether the technology is suitable enough to apply *i)* to the set of plant gene(s) associated with traits and *ii)* to induce a systemic and extended response in the plants, which would limit the production costs not only of the dsRNA but also of the nanocarrier. In conclusion, to achieve a successful Spray Gene Induced Silencing with a foliar application of RNA, several significant milestones need to be completed in conjunction with a solid knowledge of the target crop system to optimize the delivery methods and the potential addition of a nanocarrier (**Figure 2**).

## Discussion and perspectives

The availability of the foundational knowledge related to gene(s)-to-trait associations is a prerequisite for fully translating RNAi and gene editing technologies into sustainable and transformative tools for improved fruit tree production. Like other plant models, this information is mainly generated through genetic engineering studies which still are scarce in fruit trees. As the lag created by the lack of robust and tractable systems for genetic studies tends to abate, the adoption of multiplexing tools through both gene silencing and gene editing for fruit trees studies would help accelerate the acquisition of this foundational information A good example is the development of the microvine model of grapevine, which has been extensively used for reverse genetic and physiological studies (Chaïb et al., 2010; Pellegrino et al., 2019; Torregrosa et al., 2019). Major research programs developed to generate mutant collections that exist in monocots need to expand in fruit trees (Salomé, 2020; Liu et al., 2020a). Loss-of-function studies using RNAi gene silencing remain a popular tool for functional genomic purposes even with the emergence of advanced and versatile gene-editing tools. The recent development of novel RNA-targeting CRISPR/Cas effectors, like Cas13, which could take over RNAi-based silencing tools, is promising. However, collateral cleavage events of non-target RNAs with RNA-targeting Cas effectors are often observed. Its broad application remains uncertain, leaving room and time for developing more efficient RNAi-based silencing tools (Aman et al., 2018; Ai et al., 2022). Repurposing current gene-editing tools to non-editing applications like transcriptional regulation (CRISPR interference and CRISPR activation) and other aspects of epigenetic regulations is an exciting research avenue for generating mutants. Implementing such tools with new synthetic and inducible promoter systems may also offer alternate routes to prevent the hurdle of lethal mutants and provide more accurate gene-to-trait information in a spatial and temporal context. The NGS technology has enabled the generation of a massive amount of “OMICS” data in fruit trees that can increase the catalog of critical genes and their roles in primary performance traits. If the translational tools discussed in this review reach a certain level of effectiveness along with an effort to communicate the advantages stressing the non-GMO nature of these technologies, they are likely to be better accepted by the public.

Unlike RNA-based ectopic application, RNP delivery still faces major constraints, such as the clonal propagation nature of fruit trees and their recalcitrance to plant regeneration, which renders this approach a challenging alternate avenue. Efforts to develop i) protoplast regeneration protocols and ii) RNP delivery to intact regenerable tissues should be major priorities because they can significantly bolster transgene-free gene editing in fruit tree models. Nanocarriers and CPPs used in animal models must be extensively exploited in plant models for improved RNP delivery through walled regenerable tissues like the isolated examples of biolistic delivery in banana and CPP-mediated delivery in wheat microspores (Bilichak et al., 2020; Awasthi et al., 2022). Even where gene editing is performed through a transient transformation in apple (Chen et al., 2018a) and RNP-delivery in bananas (Awasthi et al., 2022), it is technically challenging to identify mutant cells. Once these milestones are achieved, the selection of edited mutants will remain tedious unless the editing efficiency rate of CRISPR/Cas is dramatically improved, making the regeneration of edited material effortless and rapid.

Pests and pathogens have conserved virulence mechanisms across host species, and the interaction mechanisms with multiple host plants often share commonalities. The knowledge from RNAi studies, such as targeting the fungal effector and RNAi fungal machinery of *Botrytis cinerea* (Wang et al., 2016; Wang et al., 2017b), the tubulin of *Drosophila suzukii* (Taning et al., 2016) and host plant’s housekeeping genes in wheat against powdery mildew fungus (Schaefer et al., 2020) should be adopted in fruit trees. Botrytis and powdery mildew are significant pathogens in many fruit trees, such as cherry, apple, and grapevine. The orthologous fungal effectors or host plant genes could be targeted through RNAi application. Though the implementation of this technology remains an issue in fruit tree orchards, a universal delivery method for all the major crops is unlikely to be developed because every crop has its plant architecture with different leaf shapes, which could be problematic for efficient delivery. The current estimates place the price-per-hectare of RNAi-based biopesticide in the range of $20-120, which relies on the cost of dsRNA synthesis and the crop production system. This corresponds to nearly 50% of the average expenses related to purchasing chemical-based products for treating the same area. Then, the broader use of this technology in the field would reduce the production cost. Future improvements in the design and the silencing efficacy of dsRNA molecules, along with the use of new carrier molecules, will favor the uptake and decrease the cost of synthesis of the active ingredients needed. Unlike gene-edited products where yield penalty can be associated with the disruption of the target genes, the RNAi technology applied to the field may offer more opportunities to manipulate traits of interest in a spatial and temporal context with lesser side effects. This technology could strengthen the confidence between producers and consumers if accepted. Overall, due to the increasing need to comply with a set of restrictive but necessary food biosafety rules, the application of biopesticides like RNAi-based products may result in better public acceptance than conventional chemical treatments. Also, by targeting traits related to yield and secondary metabolites, increased production of more nutritional food per square foot could be achieved.

## Author contributions

LD and SG contribute to the overall design of the review. SG wrote RNP delivery sections, CM, BE, and LD contributed to the RNAi-based ectopic application, and LD wrote the first section related to the current gene editing and silencing tools. All the authors contribute to the editing of the final draft.

## Funding

Part of this work is supported by [Crosscutting Program - Agricultural Innovation through Gene Editing] [grant no. 2021-67013-34555/project accession no. 1026009] from the USDA National Institute of Food and Agriculture and the Northwest Center for Small Fruits Research center (Grant # 000036).

## Conflict of interest

The authors declare that the research was conducted in the absence of any commercial or financial relationships that could be construed as a potential conflict of interest.

## Publisher’s note

All claims expressed in this article are solely those of the authors and do not necessarily represent those of their affiliated organizations, or those of the publisher, the editors and the reviewers. Any product that may be evaluated in this article, or claim that may be made by its manufacturer, is not guaranteed or endorsed by the publisher.
